# Floristic changes following the chestnut blight may be delayed for decades

**DOI:** 10.1371/journal.pone.0306748

**Published:** 2024-10-02

**Authors:** Richard Karban, Claire C. Karban

**Affiliations:** 1 Department of Entomology and Nematology, University of California, Davis, CA, United States of America; 2 Department of Ecology and Evolutionary Biology, University of Colorado, Boulder, CO, United States of America; The Clifton Institute, UNITED STATES OF AMERICA

## Abstract

A survey conducted in the 1920s, prior to the chestnut blight, indicated that chestnuts and oaks were codominant canopy species in White Oak Canyon, Shenandoah National Park, Virginia. A second survey in 1977 suggested that chestnuts were being replaced by tree species present before the blight, particularly oaks. In 2021, we resurveyed the 10 sites included in our 1977 survey and also recorded canopy and understory trees that grew above remnant chestnut sprouts. The canopy changed more substantially during the second interval (since 1977). Birch and maples were now more abundant. Hemlock declined, and oaks were less common in the canopy. In general, the trees considered as early to mid-successional have replaced oaks and hemlock. Chestnut sprouts have become much less common since 1977, presumably as repeated cycles of diebacks have weakened rootstocks. Those sites where chestnut sprouts have persisted until 2021 differed from neighboring sites without them. Chestnut sprouts were rare in sites with birch and hemlock; chestnut has persisted in locations with red oaks in the canopy and with few other understory competitors. This survey has been conducted over a longer time interval than previous studies that asked similar questions and our results suggest that changes to the forest composition following the loss of the American chestnut may be greater than previously recognized although the relative contribution of losing this codominant species is unclear.

## Introduction

There is a general consensus that anthropogenic activities have caused the climate to change over the past century [1, 2]. Other anthropogenic effects such as land use change and the introduction of exotic species have occurred simultaneously and can cause widespread mass extinctions of natives [3, 4]. Our understanding of the effects of these drivers on plant community composition and structure are less clear. A lack of quantitative data describing historical baseline conditions limits our ability to compare past plant communities with current ones [5]. As species are lost, some losses will have more wide-ranging ecological effects than others. Foundational species structure communities and stabilize conditions and processes for other species [6, 7]. In forested ecosystems, dominant trees are often foundational species, and their characteristics define forest structure and control microclimate and ecosystem processes [7]. The ecological consequences of losing these foundational species are poorly understood because the responses to these losses may be delayed; community structure and function may lag behind functional extinctions by decades.

American chestnut (*Castanea dentata*) was a co-dominant, foundational tree species (with oaks, *Quercus* spp.) in many eastern deciduous forests of North America including the Northern Blue Ridge [8]. Chestnuts grew rapidly and were tolerant of many edaphic conditions. Chestnut blight, caused by the canker fungus, *Cryphonectria parasitica*, was probably introduced to New York in 1904 [9]. By 1926 it was found throughout the native range of *C*. *dentata*. The fungus enters wounds in the bark, killing the cambium, and the infected shoot [10]. However, the fungus does not kill the roots which continue to produce new sprouts. Once a sprout reaches a height of 5–6 m, it becomes girdled and dies before it can reach the canopy or reproduce. As a result of this fungus, American chestnut has gone from a dominant canopy tree to a subordinate understory shrub [11].

The decline of American chestnut is thought to have had profound effects on terrestrial and aquatic communities [7]. For example, chestnut seeds were an important food source for many mammals (including humans), birds, and invertebrates [12, 13]. Chestnut leaves were a primary source of nutrients for stream ecosystems and those communities that depended on litter are hypothesized to have been strongly affected by the extirpation of chestnut forests [14]. Because chestnut was a widespread foundational species that disappeared over the past century, its loss can serve as a model for other foundational species that are now declining and are predicted to disappear in the future.

This study involves three surveys of the canopy trees in White Oak Canyon in Shenandoah National Park, Virginia, USA. The first survey was conducted by Lucy Braun who recorded the relative abundance of canopy tree species in the canyon before the chestnut blight [8:225]. We conducted the second and third surveys along the length of the White Oak Canyon trail in 1977 and 2021 respectively. This trail existed in the 1920s and is likely to represent the location of Braun’s survey through the narrow canyon. Pre-blight baseline data are rare, and this design enabled us to track community compositional changes occurring over nearly 100 years. Since Braun’s survey other introduced species have had large impacts on forest species including spongy moth, *Lymantria dispar*, which has impacted oaks and other susceptible trees, woolly adelgid, *Adelges tsugae*, which has decimated hemlock populations [7, 15], and anthracnose fungus which has devastated dogwoods in the understory [16].

In addition to surveys of canopy trees, we identified locations where chestnut trees had been present before the blight and compared the understory and canopy species that currently grew above these locations to determine what trees were replacing chestnut. Current chestnut sprouts and dead boles indicate locations where trees grew in the past. As mentioned above, the fungus that causes chestnut blight does not invade root tissues, and these remain alive and continue to produce sprouts [10, 11, 17]. Chestnut wood is resistant to decay so that dead standing trunks remain identifiable for decades.

This study asks these specific questions: How has the composition of the forest changed 90+ years following extirpation of a dominant species? Have changes to the forest composition been greater over the past decades compared to earlier decades? What species have filled the canopy gaps left by chestnut? Do these replacement species exhibit particular characteristics?

## Methods

### Canopy surveys

Lucy Braun published a survey of the canopy trees in the canyon that she sampled prior to the arrival of the chestnut blight around 1930 [8:225]. She did not provide precise sampling locations although she recorded 719 canopy trees of 17 species, including chestnut as a co-dominant. She described this canyon as “the most extensive area of undisturbed forest in the park.” Based on historical records of the arrival of the blight in Shenandoah National Park, this initial survey had to have been conducted prior to 1930 although the exact date of Braun’s fieldwork was not recorded, and park records and permit applications do not provide any additional information (Wendy Cass, personal communication). Parts of the canyon burned in 1930, and by 1941 chestnut was only present as sprouts and was reported to be disappearing [18].

We resurveyed 10 sites in White Oak Canyon in 1977 and again in 2021. In August 1977 we surveyed 100 randomly selected canopy trees at each of ten southeast-facing sites in White Oak Canyon along a transect between 1,050–3.500 ft elev [19]. This aspect was common and was selected as a standard to minimize effects attributable to variation in aspect. We spotted trees from the trail and decided whether to include them in our survey by flipping a coin. These were not necessarily the same precise sites that Braun surveyed. We returned to these 10 sites in June 2021 and resurveyed 100 canopy trees at each site (US Dept of the Interior Scientific Research and Collecting Permit SHEN-2021-SCI-0021). The locations of the sites were the same in the two later surveys based on maps and written descriptions although individual trees were not marked or mapped and were therefore not identical. The GPS locations of our sampling sites are shown in S1 Table.

We compared the forest community composition observed in 1977 to the community observed in 2021 using NMDS with the default Bray-Curtis dissimilarity matrix (vegan::metaMDS in R, k = 4 dimensions). Bray-Curtis is an index of dissimilarity between two communities where communities that have the same composition have a dissimilarity value of 0 and those that share no species have a value of 1 [20]. We then performed a PerMANOVA on the relative abundance of species (vegan::adonis) to assess whether community centroids were significantly different in 1977 compared to 2021 [21, 22]. We also performed a permdisp test to compare the clumping or dispersion pattern of points in the two groups in the NMDS matrix (vegan::betadisper; [22, 23]. This provides a second statistical assessment of the 1977 and 2021 communities, and is often used as a measure of species turnover, or beta diversity [24].

When the PerMANOVA test indicated that the 1977 community was different from the 2021 community, we performed an indicator species analysis to identify which tree species were associated with the community in each year (indicspecies::multipatt, [25]. In this analysis we calculated the association between each species and the year which is indicated as the point biserial correlation coefficient [26].

We categorized tree species as early, middle, or late succession following the descriptions in [27]. We placed species in these categories based on two criteria–their tolerance of shade and whether Burns and Honkala considered them as climax species. Temporal trends in species composition were evaluated by comparing the proportion of canopy trees in the early, middle, and late categories during the three surveys (pre-blight, 1977, and 2021) using a G-test of association [28].

### Associations with chestnut sprouts

In 1977, we estimated the density of chestnut sprouts and the frequency of other species that were associated with those sprouts. We selected sites that had relatively high densities of chestnut sprouts in White Oak Canyon near sites 5, 7, 8, 10 used in our floristic survey described above (S1 Table). We haphazardly selected 400 sampling points by walking 30m between sampling points and recorded whether, or not, a chestnut sprout was found within a 3m radius of each sampling point. During 20 person-hours in 1977 we surveyed 400 sampling points, 176 of which had chestnut sprouts. We returned to these same sites in 2021 and searched for any chestnut sprouts or boles that were visible in the area. We spent 40 person-hours searching and recording any visible chestnut sprouts. We spent twice the time searching for sprouts because they were much less common in 2021. After we located a chestnut sprout in 2021, we recorded the species identity of trees that were growing over any part of the space immediately above the sprout. We noted whether these other trees reached the forest canopy or were found only in the understory. We compared the tree species in the general vicinity of sprouts but not immediately above them by moving 20 m in each of the four cardinal directions from each chestnut sprout and recording the canopy and understory trees above each of these points that were greater than 2 m tall. These four samples provided an estimate of the tree species composition that was not associated with the chestnut sprout but experienced similar environmental conditions (i.e., aspect, slope, soil type, moisture). In other words, these four other sampling points served as controls which we compared to the sampling locations with chestnut sprouts.

We compared the tree species that were observed over chestnut sprouts (n = 42 sampling locations) to the overall composition of the tree community that lacked chestnut (the control locations, n = 168 locations) using NMDS with the default Bray-Curtis dissimilarity matrix (vegan::metaMDS in R, k = 3 dimensions). We then performed a PerMANOVA on the relative abundance of the species (vegan::adonis) to assess whether overstory community centroids were significantly different above chestnut sprouts and above control points [22, 23]. This calculation is based on sums of squared deviations from each data point to the control, so difference in centroid location can be confounded with the spatial patterning of points. To tease out this effect we also performed a permdisp test to compare the clumping or dispersion pattern of points in the two groups in the NMDS matrix (vegan::betadisper). This provides a second assessment of the communities above chestnut and above other surrounding vegetation.

Since the PerMANOVA and permidsp tests indicated that the assemblage of trees above chestnut sprouts was different from the controls, we used an indicator species analysis to identify those tree species that were associated with each location type: chestnut or control (indicspecies::multipatt, [24]. In this analysis we calculated the association between each species and the location type which is indicated as the point biserial correlation coefficient, corrected for unbalanced group sizes [25]. All NMDS and associated analyses were conducted in R.

## Results

### Canopy surveys

The composition of the forest had not changed much between Lucy Braun’s survey prior to the chestnut blight and the survey approximately 50 years later in 1977 (Table 1). By 1977 chestnut trees were gone from the canopy where Braun conducted her survey, but the relative abundances of other tree species were rather similar. Three of the next four most abundant species before the blight, had largely taken the place of chestnut in the canopy; *Quercus rubra*, *Acer saccharum*, and *Quercus montana* all increased in abundance in the 1977 survey. Only *Tsuga canadensis* failed to increase in 1977 relative to its abundance before the blight.

**Table 1:** Canopy composition in White Oak Canyon before and after the chestnut blight.

Species	Before the blight (Braun)	Before blight excluding chestnut	1977	2021
Castanea dentata	15.2	0	0	0
Quercus rubra (L)	15.2	17.9	21.6	13.6
Acer saccharum (L)	12.5	14.8	18.8	18.4
Tsuga canadensis (L)	12.1	14.3	11.4	2.9
Quercus montana * (L)	11.1	13.1	15.0	3.1
Quercus alba (L)	9.2	10.9	9.8	5.6
Tilia spp.* (L)	7.4	8.7	2.4	3.2
Betula lenta (M)	4.9	5.8	10.6	17.9
Liriodendron tulipifera (E)	3.3	3.9	1.0	3.9
Carya spp. (L)	2.5	3.0	3.3	2.6
Acer rubrum (E)	2.5	3.0	1.1	10.5
Fraxinus americana (E)	1.5	1.8	1.8	2.2
Ulmus rubra * (L)	1.1	1.3	0	4.8
Robinia pseudoacacia (E)	0.8	0.9	1.2	0.6
Juglans cinerea (L)	0.4	0.5	0.2	0.6
Nyssa sylvatica (M)	0.3	0.4	0.3	0.2
Juglans nigra (L)	0.1	0.1	0.1	0.1
Pinus pungens (E)	-	-	0.7	0.3
Pinus strobus (M)	-	-	0.3	0.3
Acer pennsylvanicum (M)	-	-	0.2	7.0
Sassafras albidum (E)	-	-	0.1	0.5
Crataegus spp. (E)	-	-	0.1	-
Pinus rigida (E)	-	-	-	0.8
Prunus serotina (M)	-	-	-	0.4
Platanus occidentalis (E)	-	-	-	0.3

Data are percentages of the total number of trees. The letters that follow each tree species indicate the successional stage that we assigned to it following Burns and Honkala [26] (E–early, M–middle, L–late).

• Braun [8] listed these as *Quercus prinus*, *Tilia neglecta* and *Ulmus fulva*.

The forest composition changed more dramatically between 1977 and 2021 (Table 1). The oaks that had been a major component of the forest in both the pre-blight and 1977 surveys were less common in 2021 (Table 1; *Q*. *rubra* decreased by 37%, *Q*. *montana* by 86%, *Q*. *alba* by 43% in 2021 relative to 1977). Most of the oaks that we saw during the later survey were canopy trees and there were very few *Quercus* seedlings except at the top of the canyon (personal observation). Hemlocks (*Tsuga canadensis*) were less common in 1977 than in the pre-blight survey and this decline was more noticeable in 2021 (Table 1; a 75% decline between 1977 and 2021). Birch (*Betula lenta*) and two maples (*Acer rubrum* and *A*. *pennsylvanicum*) registered large increases in 2021 compared to earlier surveys (Table 1).

**Fig 1:**
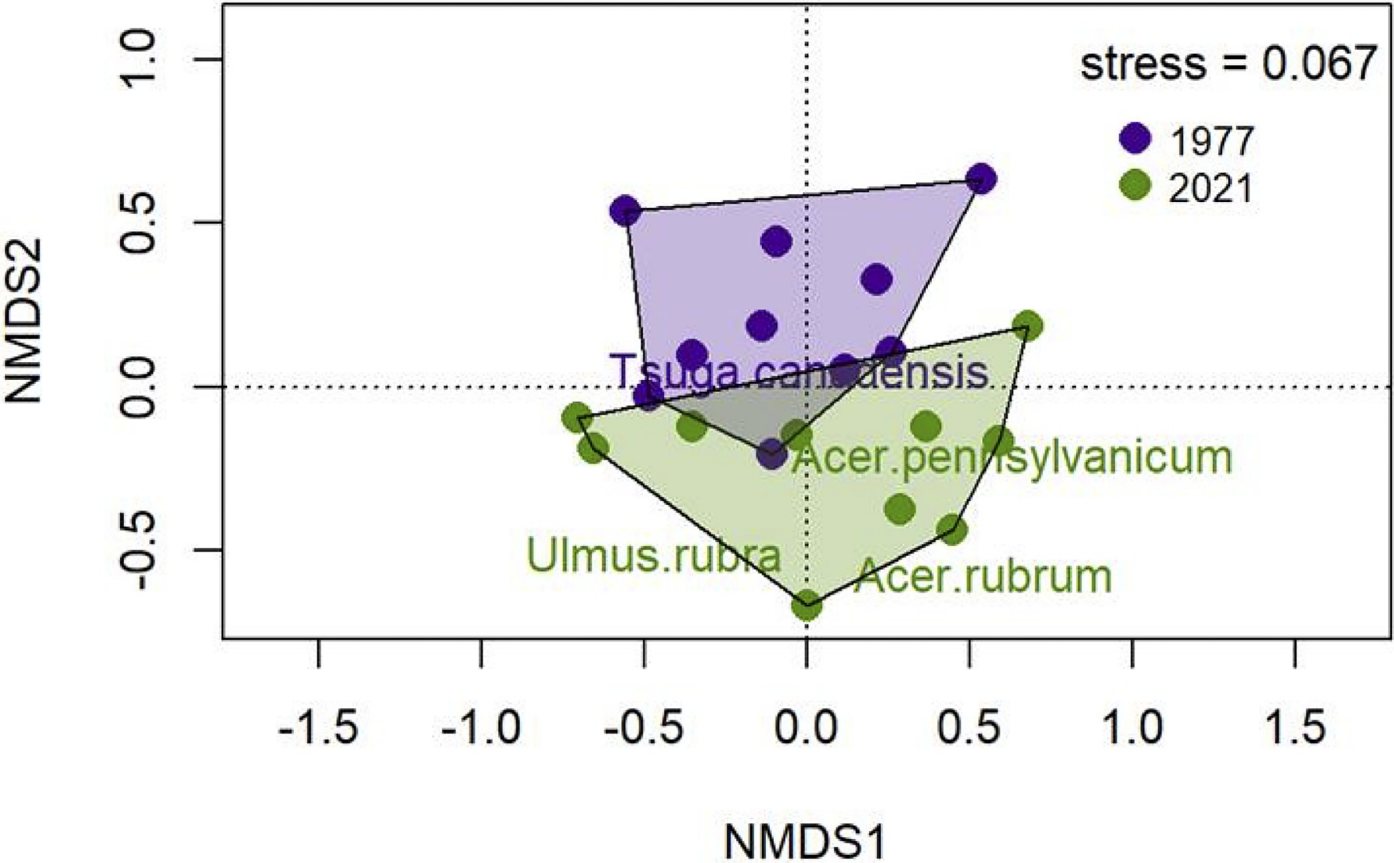
The first two of four axes of a nonmetric multidimensional scaling ordination of canopy composition for the 1977 and 2021 surveys (stress = 0.067). Hulls are drawn around each group– 1977 and 2021. The species names shown in color were significantly correlated with year in the indicator species analysis (see S4 Table).

**Fig 2:**
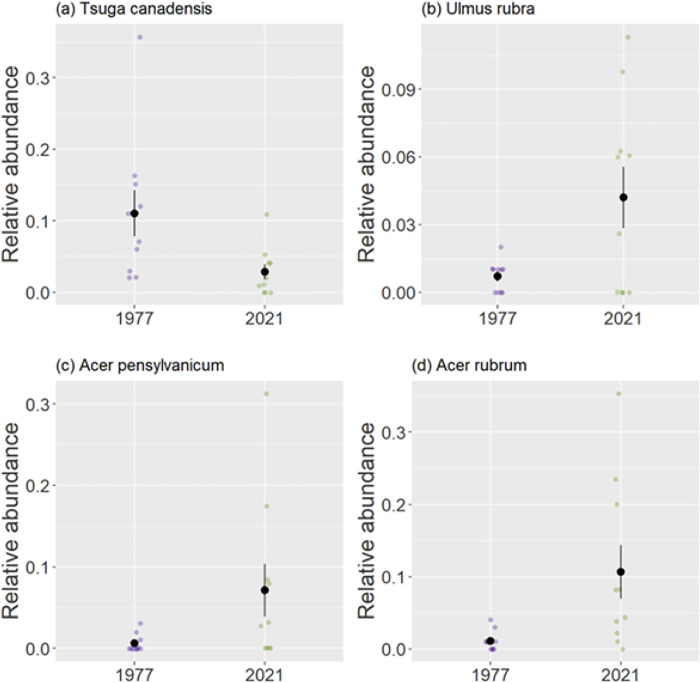
The relative abundances of tree species in canopy surveys in 1977 and 2021. Black points show mean relative abundances with standard error bars.

Changes in forest composition between 1977 and 2021 can be visualized by comparing the communities along two non-dimensional axes (Fig 1). There was relatively little overlap between the hulls that represented the two communities, particularly along axis 2. This difference in community composition was statistically significant (PerMANOVA, F _1,18_ = 2.521, group R^2^ = 0.123, P = 0.032, S2 Table), and it was not driven by dispersion (S3 Table). These shifts were represented by a significant decrease in the abundance of hemlock in 2021 compared to 1997 (*Tsuga canadensis* was significantly associated with the 1977 community [p = 0.018]; Fig 2A; S4 Table). Two maples along with slippery elm (*Ulmus rubra*) showed significant increases in relative abundances in 2021 (*Ulmus rubra*, *Acer pennsylvanicum*, and *Acer rubrum* were significantly associated with the 2021 community [*U*. *rubra*, p = 0.032, *A*. *pennsylvanicum*, p = 0.037, *A*. *rubrum*, p = 0.004]; Fig 2B–2D; S4 Table).

The successional status of the canopy changed over the time interval of these surveys (Table 2; G = 283, df = 4, P < 0.001). Early and mid-successional species were more common in the 2021 survey at the expense of later successional species. Most change occurred since 1977.

**Table 2:** Association between successional status and date of the survey.

Successional Stage	Pre-blight (<1930)	1977	2021	Total
Early	9.6	6.0	18.9	34.5
Middle	6.2	11.4	26.4	44.0
Late	84.6	82.6	55.0	222.2
Total	100.4	100.0	100.3	

Numbers are percentages of canopy trees in each category. Totals exceed 100 due to rounding error.

### Associations with chestnut sprouts

Chestnut sprouts were much less common in 2021 compared to 1977. In both years we recorded the number of sprouts that we encountered after searching in the same locations. We found 176 sprouts in 20 person-hours in 1977 and only 42 sprouts in 40 person-hours of searching in 2021.

**Fig 3:**
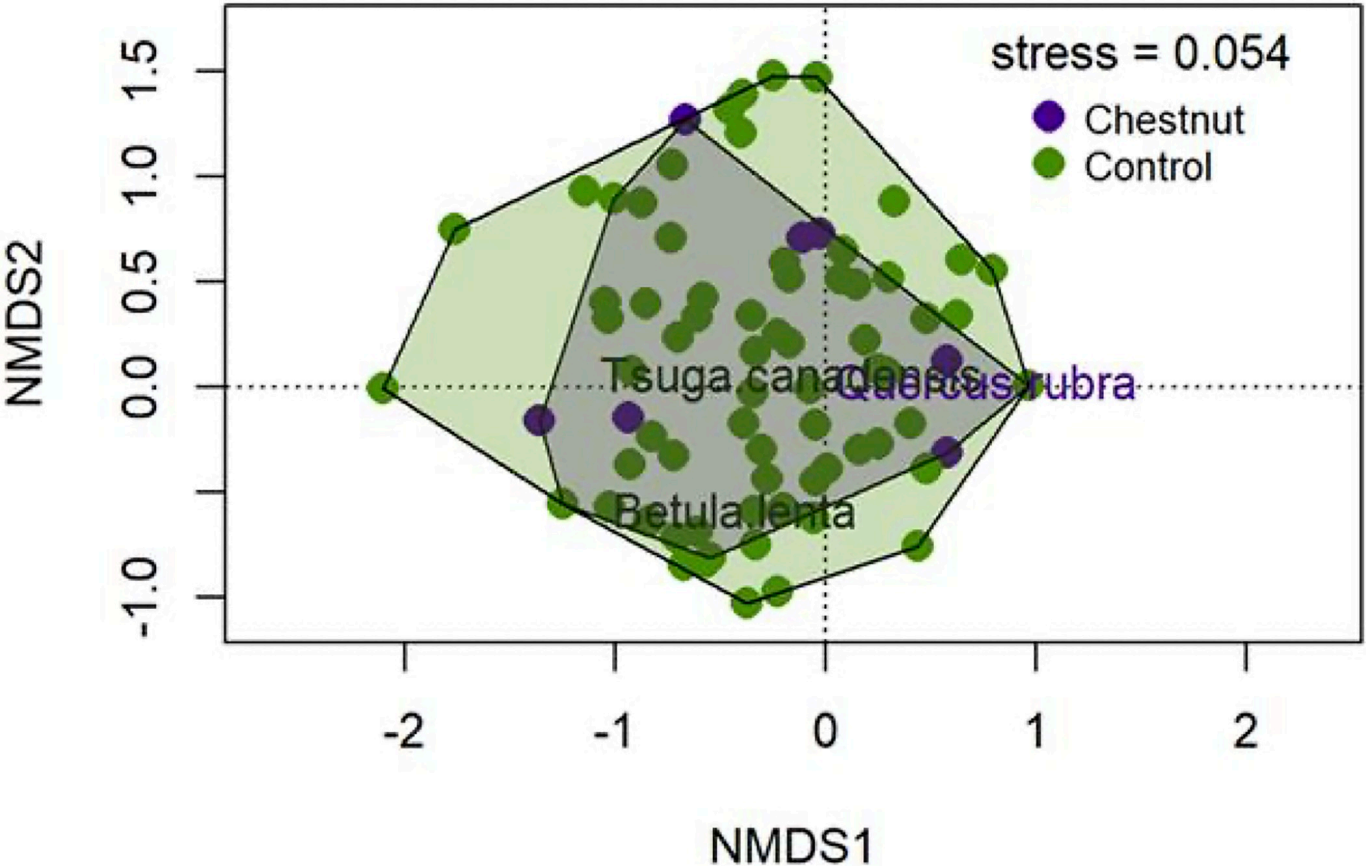
The first two of three axes of a nonmetric multidimensional scaling ordination of canopy composition above chestnut sprouts and control points (stress = 0.054). Hulls are drawn around each group–chestnut and control. The highlighted species were significantly correlated with the group in the indicator species analysis (see S5 Table).

Differences between community composition above chestnut trees and control plots are visualized along the first two NMDS axes (Fig 3). Community composition was significantly different above chestnut sprouts (PerMANOVA, F _1,207_ = 4.952, group R^2^ = 0.023, P = 0.001, S2 Table). While there was considerable overlap in the polygons, the statistical difference in community composition was likely driven by a significant difference in point dispersion between the chestnut and chestnut-free control groups (permdisp, F _1,207_ = 6.480, p = 0.012, S3 Table) rather than a difference in the polygon centroids. This suggests a different in beta diversity between areas with chestnut sprouts and those without.

**Fig 4:**
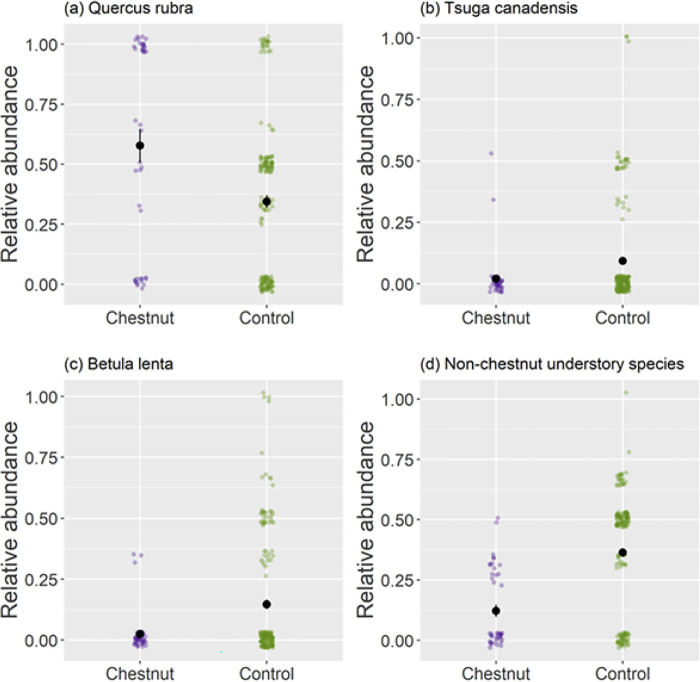
The relative abundance of species in the two treatment groups (above chestnut sprouts or chestnut-free [controls]). Larger colored points indicate outliers. Data points are overlayed as smaller black points. *Quercus rubra* (a) was significantly associated with chestnut sprouts (p = 0.003), while *Tsuga canadensis* (b) and *Betula lenta* (c) were significantly associated with chestnut-free plots (*B*. *lenta*, p = 0.002 and *T*. *canadensis*, p = 0.026). Stems in the understory of species other than chestnut (d) were negatively associated with the presence of chestnut sprouts (p = 0.001).

Chestnut sprouts were not randomly distributed throughout the forest; *Quercus rubra* was significantly associated with the presence of chestnut sprouts (p = 0.003). Locations with chestnut sprouts had almost double the number of *Q*. *rubra* compared to control points (Fig 4A). Many of the chestnut sprouts were in locations with large oak trees (including *Q*. *rubra*) in the canopy but no other trees between them and the canopy. *B*. *lenta* (p = 0.002) and *T*. *canadensis* (p = 0.026) were significantly associated with the control group (Fig 4B and 4C). In contrast with the chestnut locations, chestnut-free locations had more than twice the density of understory trees of all species. All stems other than chestnut that were in the understory were under-represented at locations that had chestnut sprouts (Fig 4D, Table 3, *X*^2^ = 15.37, df = 1, P = 0.001). In other words, chestnut sprouts tended to be found at sites that were depauperate of understory competitors.

**Table 3:** Trees growing over the canopy of the chestnut sprouts and at four locations away from each chestnut sprout (controls).

Species	Chestnut		No Chestnut (Control)	
	Canopy x 4	Understory x 4	Canopy	Understory
Q. rubra	184	8	114	5
A. saccharum	44	8	23	16
T. canadensis	0	8	8	23
Q. alba	48	0	44	0
B. lenta	4	8	21	44
A. pennsylvanicum	0	12	2	38
H. virginiana	0	24	0	28
Other	12	4	14	9
Total	292	72	226	163

Since we sampled four times as many control points as chestnut sprouts, we have multiplied the number of trees above the chestnut sprouts by four for comparison and present these normalized counts. Trees that reached the canopy were differentiated from understory trees that did not reach the canopy.

## Discussion

Other studies have examined the changes that have occurred in forests previously dominated by American chestnut. Many of these studies reported that *Quercus* species that had been codominant before the blight filled in the gaps left by dying chestnuts in New England [29], Pennsylvania [30], Virginia [31], and North Carolina [32–35]. Species other than oaks assumed dominant roles under specific climatic, disturbance, and edaphic conditions (e.g., [35, 36]). However, the current study includes a longer time frame than past work, thanks in part to the availability of accurate estimates of the historical forest composition and conditions, which is particularly hard to come by [37]. Further, we include both surveys of the composition of the forest canopy before and after arrival of the blight along with observations of the successional process in gaps created by dying chestnuts.

We previously reported that codominant species, *Quercus rubra*, *Acer saccharum* and *Quercus montana* increased in White Oak Canyon between the pre-blight survey and 1977 ([19], see also Table 1). Aside from a decline of *Tsuga canadensis*, the forest in 1977 was rather similar to the pre-blight composition without chestnut. The changes that occurred between 1977 and 2021 were more profound and included a greater representation of *Betula lenta*, *Acer rubrum*, and *Acer pennsylvanicum* (Table 1), although only the maples were significantly associated with the 2021 survey (Figs 1, 2 and S4 Table). *B*. *lenta* is shade intolerant and not considered a climax species [38]. *A*. *rubrum* is also considered as a subclimax species [39]. *A*. *pennsylvanicum* is shade tolerant but never becomes a canopy species [40]. *Ulmus rubra* was also statistically associated with the 2021 survey (Figs 1 and 2) although it was never common in our surveys. These species that have increased in recent years were responsible for a trend away from species that are characterized as later successional (Table 2). These successional categories are context-dependent and open to interpretation; however, the trend in Table 2 is sufficiently strong so that a reclassification of some of these species will not change this overall conclusion.

It is unclear why changes since 1977 were more noticeable than those that occurred previously. There are several factors that may have contributed to this pattern. 1) Successional changes occur slowly and it may have taken many decades before the trees became large enough to influence the survey counts. Rates of change tend to slow late in the secondary succession of forests [41]. Lagged responses to new environmental conditions are not unusual, particularly for long-lived organisms such as trees [42].

2) Prior to 1977 chestnut trees had disappeared from the canopy but were still common and strong competitors for resources below ground. Understory species are known to exert strong effects on the successional dynamics of canopy species [43]. By 2021, repeated cycles of resprouting and trunk death have weakened the existing chestnut sprouts and they are disappearing from the landscape. We located approximately an order of magnitude fewer sprouts in 2021 compared to the rate at which we found them in 1977. Most of the chestnut sprouts that we found in 2021 had large oaks in the overstory and fewer competitors in the understory relative to locations that lacked chestnut sprouts (Table 3).
3) Changes in other important environmental conditions have been more pronounced since 1977 than during the decades between 1930 and 1977. For example, the pace of global climate change has increased over recent decades and is predicted to accelerate for the central Appalachians [44]. Warming temperatures were found to provide the greatest benefits to *Betula lenta* and *Acer rubrum* in another study [45], and these are the species that we found had increased most dramatically since 1977. Since 1977, *Quercus montana* has shown a particularly large decline (Table 1). This species grows in exposed, dry sites with shallow soils and it may be particularly sensitive to climate change.
4) In recent decades introduced pests have decimated other important tree species in White Oak Canyon and these novel disturbances may have had synergistic effects with the chestnut blight. An outbreak of the hemlock wooly adelgid (*Adelges tsugae*) since 1988 has caused large declines to *Tsuga canadensis* which was one of the four most common canopy species in 1977 and is declining rapidly in White Oak Canyon [46, 47]. The disappearance of hemlock strongly affects the soil, microclimate, hydrology, and community and ecosystem processes where it co-occurred [7]. An outbreak of spongy moths (*Lymantria dispar*) that occurred from 1984–1995 caused considerable mortality to oak trees in this region [15, 48]. This outbreak may have contributed to the reduction of oaks in the canopy that we observed in 2021 [15].
5) Over the past century deer populations have increased markedly and this trend has intensified since 1977 in many eastern forests [49]. Overbrowsing shifts communities towards browse tolerant or resistant species and this effect has the potential to dominate other drivers of forest composition [50]. For example, overbrowsing favors maples at the expense of oaks [51].
6) Similarly, fire suppression can favor birch and maples at the expense of oaks, similar to the pattern observed here [52, 53]. Since 1930, there have been no fires in white oak canyon and less fire-adapted species are predicted to continue to increase in frequency as canopy trees die unless the fire regime changes. This hypothesis highlights the importance of sensitivity to fire and downplays the role of shade tolerance as drivers of forest succession [53]. Chestnut was one of the most flammable species in this ecosystem so losing it may have further reduced the frequency of fires [54].
7) The results could have been caused by sampling different areas and individual trees over time. However, this explanation is unlikely to produce the pattern that we observed. We lack the precise locations where Braun sampled prior to the blight but we have detailed maps and descriptions that we used in 1977 and we were able to return to those spots to resurvey in 2021. Since the survey results were relatively similar between pre-1930 and 1977 but relatively different between 1977 and 2021, imprecision about sampling sites would not be expected to cause these results.

Chestnuts have persisted at this site despite not reproducing for nearly 100 years; they are now being replaced. It is remarkable that they have endured as an understory species for this long. In virtually all sites where we observed chestnut sprouts, other species have now filled in the canopy openings above them. Persisting chestnut sprouts were found primarily at microsites with relatively high light levels [55, 56]. Since 1977, they have only survived in microsites in White Oak Canyon with relatively deep soils that lack high densities of understory competitors or trees that produce deep shade (Figs 3 and 4). Sprouts were often found at sites with mature red oaks in the canopy and few understory or early and mid-successional trees.

Many factors influence the composition of the forest canopy. It is difficult to confidently evaluate the ecological drivers of the changes that long-term surveys such as ours have revealed. Whereas existing co-dominant oaks seemed to be replacing chestnuts in our earlier survey, we now observed a greater abundance of earlier successional species. Although the blight rapidly eliminated chestnut in the canopy as a co-dominant almost a century ago, the effects of this and other contemporaneous changes on community structure have only become apparent in recent decades. The increase in early successional species coupled with a decrease in oaks is likely to be accompanied by changes in life history, structural, and energetic properties of the entire forest. Other changes to the climate and novel invasive pests in addition to the chestnut blight fungus are also likely to have had significant effects on the species composition that we observed during this more recent survey. It is important to increase our efforts to monitor the changes that occur as multiple anthropogenic disturbances play out over time.

Knowledge about the composition of the forest before the chestnut blight could inform efforts to restore this community. Pollen cores suggest that chestnut was common at some sites as early as 9400 yrs BP and charcoal remains from Tennessee indicate that chestnut was most abundant there 1000–300 yrs BP [57]. Native Americans likely facilitated the spread and abundance of chestnuts since they valued it as a source of food [58]. In the centuries before European colonization, the abundance of chestnut appeared relatively stable at many sites [59]. Chestnut resprouts well after cutting and it is likely that chestnut was more dominant in the 18^th^ and 19^th^ centuries than it had been previously [58, 59]. Fires were common during this period of European influence although the relationship between fire frequency and the abundance of chestnut is controversial [54, 58]. Birch and maple are thought to have also increased during the post-colonization period. It becomes unclear what a realistic target for restoration should be–pre-colonization communities, those that immediately preceded the blight, or those predicted to be adapted to future climates.

Since chestnut was a foundational species that was dominant over large areas of eastern North America, its disappearance is also likely to have far-reaching effects on the other inhabitants of these forests and on ecosystem processes (described in detail by [7]. Most significantly, this study suggests that many of the structural and functional changes may not be detectable for decades after the foundational species become actually or functionally eliminated. While we and many other workers concluded that the extirpation of chestnut across eastern North America had relatively small effects on the floristic composition of the remaining forest (see references in the introduction and above), that conclusion may have been premature if more profound changes require longer time frames to become apparent. Future losses of other foundational species may also take decades or centuries to play out.

## Supporting information

S1 Table Location of survey sites in White Oak Canyon. (DOCX)

S2 Table PerMANOVA results from Bray-Curtis dissimilarities using abundance data for forest community above chestnut sprouts and at control points. (DOCX)

S3 Table PERMDISP results from Bray-Curtis dissimilarities using abundance data for forest community above chestnut sprouts and at control points. (DOCX)

S4 Table Results of an analysis of indicator species for species associated with forest communities in 1977 and 2021. Significant species are bolded.
(DOCX)

S5 Table Results of an analysis of indicator species for species associated with chestnut sprouts and chestnut-free controls locations. Significant species are bolded.
(DOCX)
