# Supplementary material for: Floristic changes following the chestnut blight may be delayed for decades
Source: PLoS One. 2024 Oct 2;19(10):e0306748. doi: 10.1371/journal.pone.0306748 (PMC11446440; doi:10.1371/journal.pone.0306748)
Supplement: S1 Table — (DOCX) [file pone.0306748.s001.docx]

Table S1. Location of survey sites in White Oak Canyon.

| Site | Elevation m | Latitude | Longitude |
| --- | --- | --- | --- |
| 1. NE of trail w/in park boundary | 324 | 35.541 | -78.351 |
| 2. rocky above lowest falls | 457-474 | 38.556 | -78.352 |
| 3. middle falls | 539-599 | 38.555 | -78.357 |
| 4. upper falls | 790 | 38.564 | -78.364 |
| 5. along creek | 813-855 | 38.566 | -78.366 |
| 6. below rock ford | 918-922 | 38.573 | -78.369 |
| 7. btwn limberlost & horse trail | 988-1005 | 38.577 | -78.353 |
| 8. above wooden bridge | 1013 | 38.581 | -78.380 |
| 9. below parking on trail | 1032-1036 | 38.584 | -78.381 |
| 10. S of WOC parking | 1032 | 38.585 | -78.383 |
