# Supplementary material for: Floristic changes following the chestnut blight may be delayed for decades
Source: PLoS One. 2024 Oct 2;19(10):e0306748. doi: 10.1371/journal.pone.0306748 (PMC11446440; doi:10.1371/journal.pone.0306748)
Supplement: S2 Table — (DOCX) [file pone.0306748.s002.docx]

Table S2. PerMANOVA results from Bray-Curtis dissimilarities using abundance data for forest community above chestnut sprouts and at control points. Df – degrees of freedom; Sum Sq – sum of squares; p-value based on 999 permutations.

| Comparison | Variable | Df | Sum Sq | Model F | R^2^ | P |
| --- | --- | --- | --- | --- | --- | --- |
| Chestnut – control | chestnut | 1 | 1.479 | 4.952 | 0.023 | 0.001 *** |
|  | residuals | 207 | 61.815 | - | 0.977 | - |
|  | total | 208 | 63.294 | - | 1.000 | - |
| 1977 – 2021 | year | 1 | 0.319 | 2.521 | 0.123 | 0.032 * |
|  | residuals | 18 | 2.277 | - | 0.877 | - |
|  | total | 19 | 2.596 | - | 1.000 | - |
