# Supplementary material for: Floristic changes following the chestnut blight may be delayed for decades
Source: PLoS One. 2024 Oct 2;19(10):e0306748. doi: 10.1371/journal.pone.0306748 (PMC11446440; doi:10.1371/journal.pone.0306748)
Supplement: S3 Table — (DOCX) [file pone.0306748.s003.docx]

Table S3. PERMDISP results from Bray-Curtis dissimilarities using abundance data for forest community above chestnut sprouts and at control points. Df – degrees of freedom; Sum Sq – sum of squares

| Comparison |  | Df | Sum Sq | F | P |
| --- | --- | --- | --- | --- | --- |
| Chestnut – Control | groups | 1 | 0.395 | 6.481 | 0.012* |
|  | residuals | 207 | 61.815 | - | - |
| 1977 – 2021 | Groups | 1 | 0.017 | 1.828 | 0.193 |
|  | residuals | 18 | 0.168 | - | - |
