# Supplementary material for: Floristic changes following the chestnut blight may be delayed for decades
Source: PLoS One. 2024 Oct 2;19(10):e0306748. doi: 10.1371/journal.pone.0306748 (PMC11446440; doi:10.1371/journal.pone.0306748)
Supplement: S4 Table — Significant species are bolded. (DOCX) [file pone.0306748.s004.docx]

Table S4. Results of an analysis of indicator species for species associated with forest communities in 1977 and 2021. Significant species are bolded.

| Species | Year | Pt. biserial corr. coeff. | p-value |
| --- | --- | --- | --- |
| **Hemlock** | **1977** | **0.479** | **0.0179** |
| Chestnut oak | 1977 | 0.411 | 0.0625 |
| Red oak | 1977 | 0.322 | 0.1740 |
| Black locust | 1977 | 0.302 | 0.2790 |
| Tupelo | 1977 | 0.295 | 0.2611 |
| Table mountain pine | 1977 | 0.184 | 0.4778 |
| Hickory spp. | 1977 | 0.181 | 0.4576 |
| White oak | 1977 | 0.067 | 0.7933 |
| Sugar maple | 1977 | 0.196 | 1.000 |
| **Red maple** | **2021** | **0.453** | **0.0037** |
| **Slippery elm** | **2021** | **0.484** | **0.0323** |
| **Striped maple** | **2021** | **0.393** | **0.0372** |
| Birch | 2021 | 0.393 | 0.0928 |
| Butternut | 2021 | 0.302 | 0.3299 |
| Yellow poplar | 2021 | 0.273 | 0.3174 |
| White ash | 2021 | 0.175 | 0.5133 |
| Black walnut | 2021 | 0.128 | 0.6289 |
| Basswood | 2021 | 0.140 | 1.000 |
