# Supplementary material for: Floristic changes following the chestnut blight may be delayed for decades
Source: PLoS One. 2024 Oct 2;19(10):e0306748. doi: 10.1371/journal.pone.0306748 (PMC11446440; doi:10.1371/journal.pone.0306748)
Supplement: S5 Table — Significant species are bolded. (DOCX) [file pone.0306748.s005.docx]

Table S5. Results of an analysis of indicator species for species associated with chestnut sprouts and chestnut-free controls locations. Significant species are bolded.

| Species | Group | Pt. biserial corr. coeff. | p-value |
| --- | --- | --- | --- |
| **Red oak** | **Chestnut** | **0.261** | **0.0026** |
| White oak | Chestnut | 0.057 | 0.5554 |
| Sugar maple | Chestnut | 0.043 | 0.6439 |
| Chestnut oak | Chestnut | 0.016 | 0.9547 |
| **Birch** | **Control** | **0.282** | **0.0015** |
| **Hemlock** | **Control** | **0.211** | **0.0258** |
| Striped maple | Control | 0.181 | 0.0516 |
| Red maple | Control | 0.156 | 0.1745 |
| Hickory | Control | 0.077 | 0.6841 |
| Elm | Control | 0.073 | 0.8401 |
| Witch hazel | Control | 0.031 | 0.8165 |
| Eastern white pine | Control | 0.055 | 1.0000 |
| Locust | Control | 0.055 | 1.0000 |
| Tupelo | Control | 0.055 | 1.0000 |
| Ash | Control | 0.17 | 1.0000 |
